# Linking biosynthetic and chemical space to accelerate microbial secondary metabolite discovery

**DOI:** 10.1093/femsle/fnz142

**Published:** 2019-06-28

**Authors:** Sylvia Soldatou, Grimur Hjorleifsson Eldjarn, Alejandro Huerta-Uribe, Simon Rogers, Katherine R Duncan

**Affiliations:** 1 Department of Chemistry, University of Aberdeen, Aberdeen, UK. AB24 3UE; 2 School of Computing Science, University of Glasgow, Glasgow, UK. G12 8RZ; 3 Strathclyde Institute of Pharmacy and Biomedical Sciences, University of Strathclyde, Glasgow, UK. G4 0RE

**Keywords:** secondary metabolites, specialised metabolites, biosynthetic gene clusters, genome mining, comparative metabolomics

## Abstract

Secondary metabolites can be viewed as a chemical language, facilitating communication between microorganisms. From an ecological point of view, this metabolite exchange is in constant flux due to evolutionary and environmental pressures. From a biomedical perspective, the chemistry is unsurpassed for its antibiotic properties. Genome sequencing of microorganisms has revealed a large reservoir of Biosynthetic Gene Clusters (BGCs); however, linking these to the secondary metabolites they encode is currently a major bottleneck to chemical discovery. This linking of genes to metabolites with experimental validation will aid the elicitation of silent or *cryptic* (not expressed under normal laboratory conditions) BGCs. As a result, this will accelerate chemical dereplication, our understanding of gene transcription and provide a comprehensive resource for synthetic biology. This will ultimately provide an improved understanding of both the biosynthetic and chemical space. In recent years, integrating these complex metabolomic and genomic data sets has been achieved using a spectrum of manual and automated approaches. In this review, we cover examples of these approaches, while addressing current challenges and future directions in linking these data sets.

## Teaser

This review covers advances in methods used to link microbial genes to the chemistry they encode

## INTRODUCTION

Recent improvements in the identification of BGCs has revolutionised our capacity to understand secondary metabolite production. Over the last few years, there has been a significant effort to link genomic data to secondary metabolite data for microorganisms, in particular bacteria. The first section of this review focuses on the creation of both biosynthetic and chemical data sets used for this purpose. The term *linking* in this review will cover all aspects of associating BGCs with the metabolite they encode. This linking process is divided into two main sections. The first is *targeted approaches to linking*, comprising the subsections, *targeted genome mining linking*, whereby strains are selected based on BGC information for further chemical analysis and *multi-targeted linking*, encompassing genome mining with bioactivity, metabolomics and proteomics approaches. The second is automated approaches, which is further divided into correlation-based and feature-based. Correlation-based approaches identify putative links via correlation of strain inclusion in clusters of spectra and BGCs. Feature-based approaches score individual spectra against individual BGCs based on shared properties. At the same time, we appreciate that studies rarely fall into these binary categories and that in reality, linking is often a spectrum using both approaches. In this review, we have selected recent studies to exemplify the discovery angle of each approach. We conclude with examples of experimental validation of these links through synthetic biology methods and a section on current challenges.

## BIOSYNTHETIC AND CHEMICAL DATA SETS FOR LINKING

This section covers how data sets are made from the prediction of BGCs and detection of metabolites. This is a crucial step as the quality of the data sets will directly impact the success of linking across data sets. Predicting BGCs from bacterial genomes is a fairly mature discipline. Tools such as antiSMASH (Blin *et al*. 2017) and SMURF (Khaldi *et al*. 2010) provide BGC predictions by matching curated statistical models based on sequences of protein family domains (PFAM domains) to genomic sequences (Coggill, Finn and Bateman 2008). Such techniques typically exhibit high specificity (low numbers of false positives) at the expense of low sensitivity (high numbers of false negatives). For users wishing higher sensitivity, more speculative algorithms such as ClusterFinder (Cimermancic *et al*. 2014), MIDDAS-M (Umemura *et al*. 2013) or MIPS-GC (Umemura, Koike and Machida 2015) exist. An in-depth review of BGC detection is beyond the scope of this article – please refer to Chavali and Rhee 2018 (Chavali and Rhee 2018) for a detailed review – as are the community-driven comparative metabolomics platforms such as molecular networking, based on tandem-mass spectrometry data (Wang *et al*. 2016) and data bases such as NP Atlas (www.npatlas.org). While cross-referencing data bases has been the status quo for secondary metabolite identification, facilitating this through automation would greatly accelerate discovery.

## TARGETED LINKING

### Targeted genome mining approaches to linking

**Figure 1.**
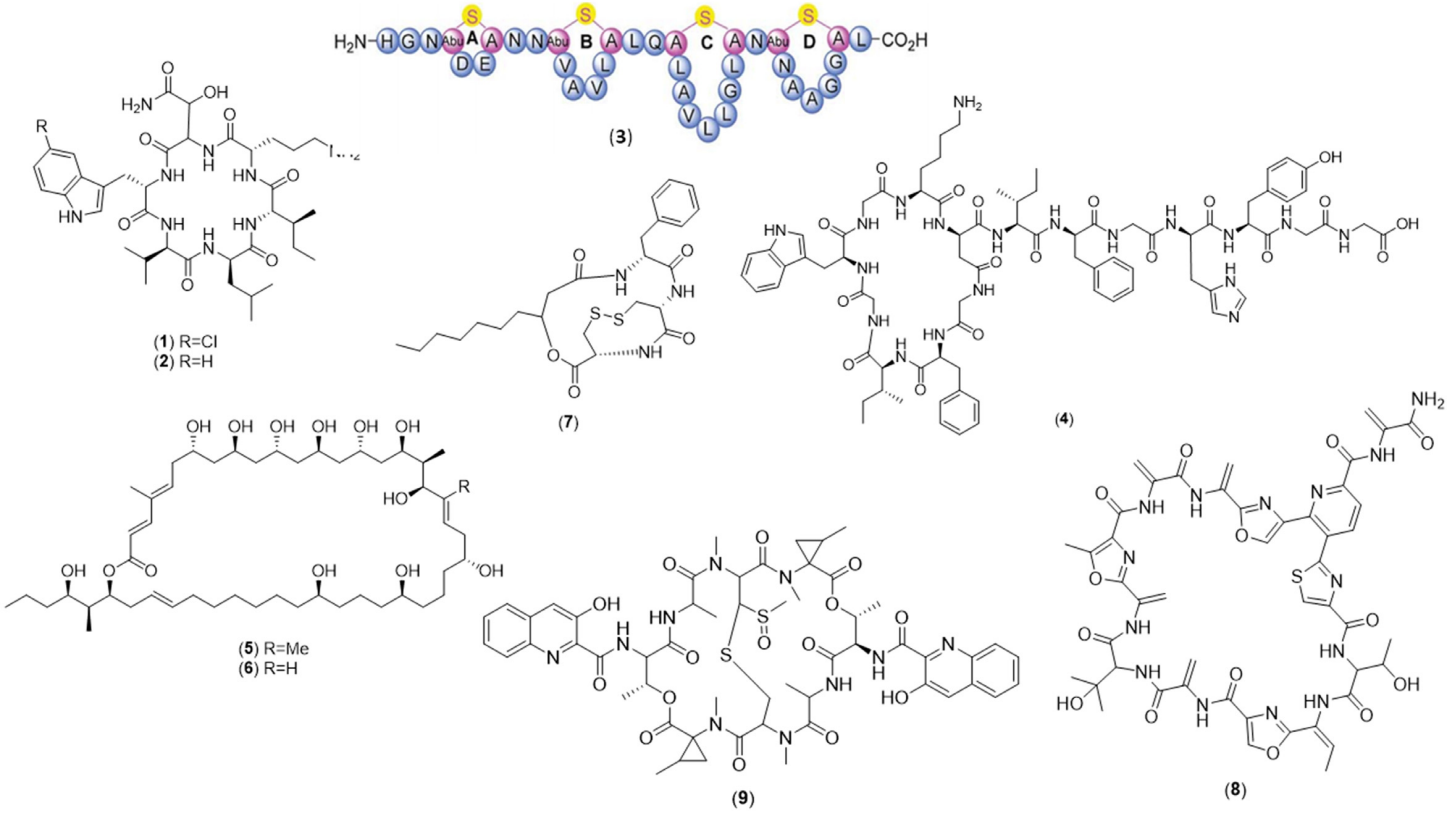
Chemical structures discovered as a result of manual linking of gene clusters and metabolites, including curacomycin (1), dechlorocuracomycin (2), tikitericin (3), ulleungdin (4), lacunalide A and B (5, 6), sunshinamide (7), geninthiocin B (8) and retimycin A (9).

In this section, strains prioritised for chemical investigation based on genome mining information, for example the presence of specific BGCs, will be discussed. This linking is often a manual process and requires specialist biosynthetic and chemical knowledge. The first step involves the dereplication, or strain prioritisation (or strain elimination due to the presence of previously discovered metabolites), within complex biosynthetic and chemical data sets. Dereplication has been greatly aided by the data analysis platforms mentioned previously, resulting in new metabolite discovery (**Fig**.**1**) (Duncan *et al*. 2015; Kaweewan *et al*. 2017; Schneider *et al*. 2018; Son *et al*. 2018; Ueoka *et al*. 2018; Xu *et al*. 2018). For example, two new peptides, the halogenated **curacomycin (1)** and its dechlorinated derivative **dechlorocuracomycin (2)**, produced by *Streptomyces curacoi* and *Streptomyces noursei* respectively, were discovered through a genome mining approach using antiSMASH to identify the presence of tryptophan halogenase genes in proximity to a nonribosomal peptide synthetase (NRPS) BGC (Kaweewan *et al*. 2017). Further genomic investigation of *S. curacoi* led to the discovery of an additional new cytotoxic peptide, curacozole, produced by a gene analogous to that of curacomycin (Kaweewan *et al*. 2019). Using a similar approach, the novel lanthipeptide **tikitericin (3)** was isolated from a thermophilic bacterium, *Thermogemmatispora* sp. by detecting a lanthionine synthetase, homologous to a class II lanthipeptide BGC (Xu *et al*. 2018). The putative BGC consisting of 10 genes encoding a new lasso peptide was observed through genome mining of the rare actinomycete *Actinokineospora spheciospongiae* and led to the isolation of actinokineosin, a new peptide with promising antibacterial activity (Takasaka *et al*. 2017). In another study, the product of the lasso peptide BGC *uld* from the genome of *Streptomyces* sp. KCB13F003 was targeted using a One Strain Many Compounds (OSMAC) approach, in which each strain was grown in multiple media in an attempt to elicit a wide range of BGC expression, resulting in the isolation of the new metabolite **ulleungdin (4)** (Son *et al*. 2018). Recently, a plant-associated *Gynuella sunshinyii* strain was prioritised based on the presence of several unassigned BGCs and six *trans*-AT PK clusters. Further investigation led to the isolation of four metabolites of which three, the polyketides **lacunalide A and B (5, 6)** and the cyclodepsitripeptide **sunshinamide (7)**-represented novel scaffolds. Further manual genome mining resulted in two ergoyne analogues, ergoyne A and B, revealing the importance of complementing automated genome mining with manual curation (Ueoka *et al*. 2018).

### Multi-targeted linking

In the last five years, comparative metabolomics has been linked with comparative genome mining, proteomics and bioactivity to accelerate discovery. For example, genome mining of a lichen-associated *Streptomyces* sp. with metabolites bioactive against *Bacillus subtilis* revealed a BGC encoding a lantibiotic. By connecting the inactivation of the BGC to the loss of the observed bioactivity, a new 35-membered macrocyclic thiopeptide antibiotic **geninthiocin B (8)** was isolated (Schneider *et al*. 2018). Natural product *proteomining* – a quantitative proteomics platform – was introduced by Gubbens *et al*., for the identification of BGCs for targeted secondary metabolites by applying the OSMAC approach, metabolomics and quantitative proteomics. This approach allowed correlations between quantitative metabolomics or bioactivity data and protein expression profiles. A new juglomycin derivative was isolated from a soil-isolated *Streptomyces* sp. by applying this quantitative multi-omics approach (Gubbens *et al*. 2014).

Using pattern-based BGC genome mining combined with comparative metabolomics through molecular networking, the relationship between BGCs and the corresponding metabolites across 35 *Salinispora* strains was assessed. This resulted in an uncharacterised PKS BGC being linked to the previously reported metabolite arenicolide A, in addition to linking an uncharacterised NRPS BGC (NRPS40) to **retimycin A (9)**, a new quinomycin-like depsipeptide (Duncan *et al*. 2015). The increasing complexity and scale of these combined data sets, often consisting of tens to thousands of both genomes and spectra, has resulted in the need to automate this process.

## AUTOMATED LINKING

Two major tracks can be observed in the automated linking of BGCs to secondary metabolites. The first approach, feature-based linking, involves linking chemical features predicted from genomic information. For example, neutral losses indicative of amino acid residues, with the observed metabolomic data. The second approach, correlation-based linking, makes use of data sets where genomic and metabolomic data are available for a large number of strains. Related BGCs assembled into *gene cluster families (GCFs)* can be correlated with spectra belonging to *molecular families (MFs)* based on the occurrence of their source strains across the data set, with the assumption that true links would have high source strain correlations.

### Feature-based linking

**Figure 2.**
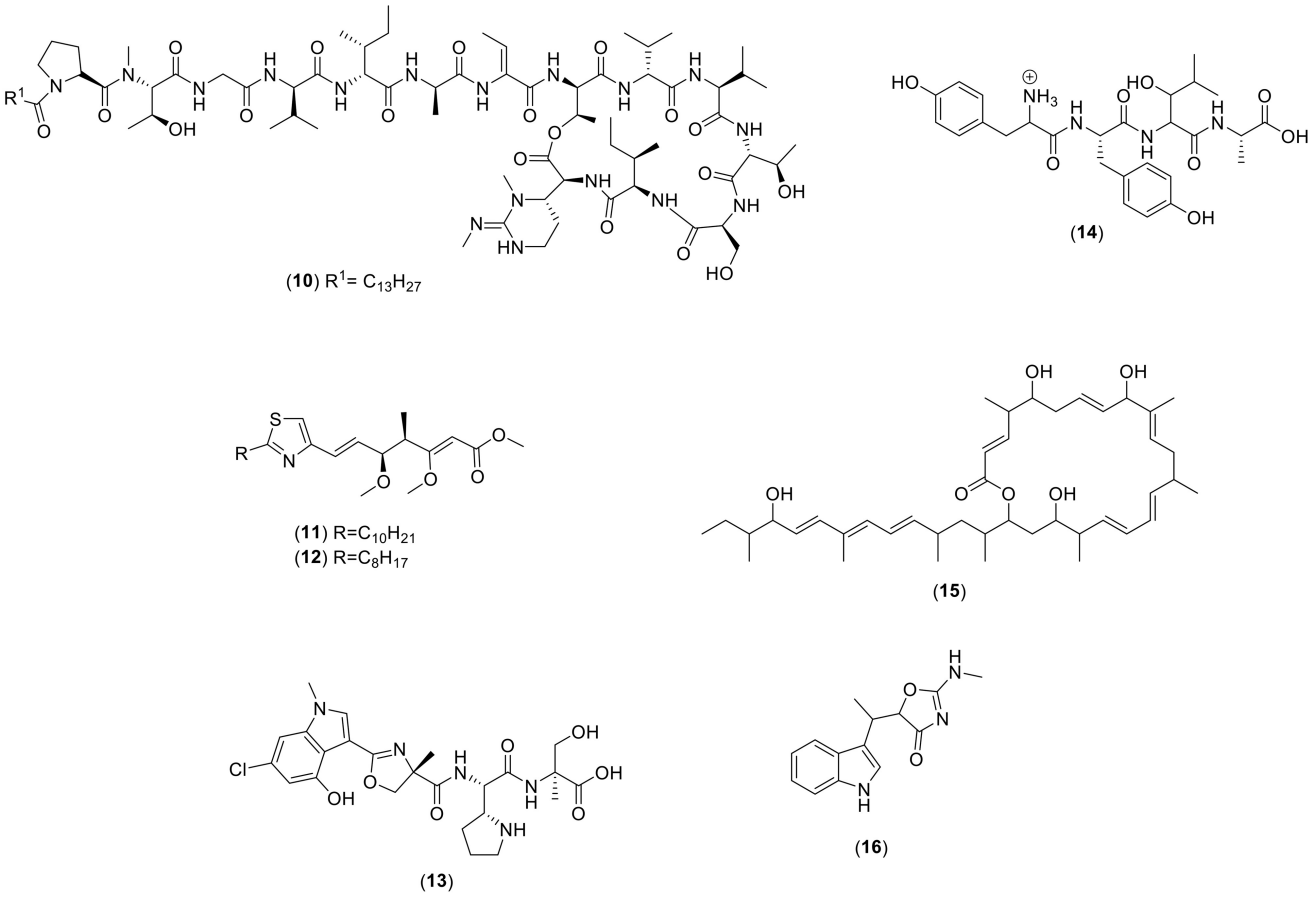
Chemical structures discovered as a result of automated linking of gene clusters and metabolites, including stendomycin I (10), fulvuthiacene A and B (11, 12), tambromycin (13), tyrobetaine (14), macrobrevin (15), and indolmycin (16).

A fruitful approach for linking BGCs to secondary metabolites has been to predict the structural properties of the molecules based on genomic information to directly detect corresponding features in mass spectra (**Fig**.**2**). For example, tools such as SANDPUMA (Chevrette *et al*. 2017) can predict substrate specificity for adenylation domains in NRPS BGCs. These predictions can be matched to amino acid residue-derived MS/MS fragmentations. This approach, using molecular properties predicted from genomic information to guide the search in chemical space, termed *peptidogenomics* by Kersten *et al*. (Kersten *et al*. 2011), has been used to link peptidic natural product BGCs to metabolites. These include **stendomycin I** (**10**) and the ribosomal lantipeptide. A similar technique has been described by Panter *et al*. (Panter, Krug and Müller 2019) for polyketides, which facilitated the discovery and structure elucidation of **fulvuthiacene A** and **B** (**11**, **12**). Other examples of tools intended to predict detectable features from genomic information include RODEO (Tietz *et al*. 2017), which is focused on RiPPs, although this has not yet been integrated with mass spectrometry data; PRISM (Skinnider et al., 2015, 2017), which focuses on NRPs and type I and II PKSs but is limited to LC-MS (but not LC-MS/MS data); GNP (Johnston *et al*. 2015), which links NRPS and PKS BGCs to LC-MS/MS spectra, Pep2path (Medema *et al*. 2014), which focuses on peptidic natural products, RippQUEST (Mohimani *et al*. 2014a) and NRPquest (Mohimani *et al*. 2014b), which detect RiPP and NRPS BGCs, respectively, and predict possible fragmentation patterns for their products.

Also relevant are tools that have been developed for dereplication. For example, DEREPLICATOR (Mohimani et al., 2017, 2018) predicts possible fragmentation patterns for peptidic natural products. By linking spectra to peptides, and identifying the BGCs responsible for the production of those peptides in databases such as MIBiG (Medema *et al*. 2015), similar BGCs from the organism can be tentatively linked to the spectra. This approach was used, for example, in Mohimani *et al*. 2018 (Mohimani *et al*. 2018), to link the polyketide antibiotic C_35_H_56_O_13_, which is structurally similar to chalcomycin, to its producing BGC.

### Correlation-based linking

Another major approach is based on matching patterns of source strain occurrence between GCFs and MFs. The assumption that similar BGCs in different strains will produce similar molecules can be used to compute a score for the link between a GCF and a MF. This builds upon early work by Lin and co-workers (Lin, Zhu and Zhang 2006) on clustering homologous BGCs (structurally similar clusters that have a shared ancestry), which was then verified by comparison with known clusters of homologous genes. Later work on clustering BGCs (Cimermancic *et al*. 2014; Doroghazi *et al*. 2014; Navarro-Muñoz *et al*. 2018) has usually involved explicit verification of the GCF, i.e. that the BGCs being grouped together are, in fact, producing related metabolites, by heterologous expression or gene knockout, but follow a similar pattern of defining novel distance functions between BGCs and constructing a clustering based upon these distances. The distances are usually defined at least partly in terms of the composition of BGCs by protein family domains, (Lin, Zhu and Zhang 2006; Navarro-Muñoz *et al*. 2018) but are often composed of multiple factors which can include everything from direct sequence similarity (Navarro-Muñoz *et al*. 2018) to the output score of sequence alignment algorithms (Doroghazi *et al*. 2014).

The clustering of spectra into Molecular Families by molecular networking is incorporated into tools such as GNPS (Wang *et al*., 2016). The similarity between any two spectra in a data set is computed using a modified cosine similarity score, to account for certain structural modifications; as a result, two spectra are taken to belong to the same MF if their score exceeds a user-defined threshold.

Once the BGCs have been clustered based on the distance measurements, the shared source strains of GCFs and MFs can be used as a starting point to correlate between BGCs and products (Doroghazi *et al*. 2014; Goering *et al*. 2016) in an approach known as *metabologenomics*. A linking score between a GCF and a MF is computed, which is dependent on the degree of strain overlap, penalising strains being present in one side (GCF or MF) and not the other. Since the presence of a BGC in a strain does not guarantee that it will be active in all circumstances (*cryptic* BGCs), this penalty is often asymmetric, with a low penalty applied for strains that contribute to the GCF but not to the spectra, while strains that contribute to the spectra and not to the GCF are highly penalised. A new chlorinated antiproliferative compound named **tambromycin** (**13**) was discovered by applying this approach to a set of 178 actinomycete strains (Goering *et al*. 2016). Moreover, a new class of natural products and their BGC were discovered when metabologenomics was combined with molecular networking. As a result, six **tyrobetaines** (**14**) bearing an unusual N-terminal trimethylammonium were identified and their BGC was confirmed through heterologous expression (Parkinson *et al*. 2018). This approach was also used by McClure *et al*. to link the rimosamide family of natural products to their corresponding BGCs (McClure *et al*. 2016).

### Hybrid approaches

Even though we have described two distinct approaches, they are not mutually exclusive. Clustering BGCs can be used in conjunction with previously established links to infer the product from the known link to the other BGCs in the cluster (Nguyen *et al*. 2013). This can also be done with databases of known BGCs, such as MIBiG, to determine which BGCs are likely to produce already known compounds (Helfrich *et al*. 2018). Clustering can therefore complement the matching of BGCs and metabolites based on predicted spectral features.

Similarly, the mutual strain information between a cluster of BGCs and a metabolite is not enough on its own to establish a correspondence between the two, especially for novel secondary metabolites. Instead, the strain content has been used to prioritise the potential links for further verification. This verification has, for instance, taken the form of predictions of common structural elements of the products. These have then been searched for in the metabolomic data (Parkinson *et al*. 2018). For example, the creation of knockout strains was used to verify the linking of **macrobrevin** (**15**) to its BGC, or the correspondence of parts of the BGCs with known parts of the pathway for the product from other organisms (Helfrich *et al*. 2018). An example of the last approach is the discovery of **indolmycin** (**16**) (Maansson *et al*. 2016), where publicly available databases and machine learning were used to create an integrated mining approach for linking gene clusters, biosynthetic pathways and secondary metabolites for 13 closely related strains of *Pseudoaltreomonas luteoviolacea*. In these strains, close to 10% of the total genes encode for secondary metabolites. This percentage is considerably higher compared to studies conducted on other *Pseudoaltreomonas* strains (Médigue *et al*. 2005; Thomas *et al*. 2008) and is corroborated by the high degree of chemical complexity reported in Maansson *et al*. 2016 as only 2% of the molecular features were shared between the investigated strains. Indeed, novel analogues of thiomarinols were detected in the molecular network of strains that were characterised as biosynthetically diverse.

## VALIDATION OF LINKS

In this section, we focus on approaches to experimentally validate links between a BGC and a secondary metabolite using synthetic biology techniques, such as genetic manipulation of BGCs. One of the most common approaches to validate the link between BGCs and metabolite is heterologous expression, the experimental details of which are outside the scope of this review. The reader is referred to Huo *et al*. for a detailed description of heterologous expression of bacterial secondary metabolite pathways (Huo *et al*. 2019).

The advent of new methods such as CRISPR/Cas9-based editing (Tao *et al*. 2018), λ-red mediated recombination (Gust *et al*. 2003), overexpression of positive regulators (Bergmann *et al*. 2010) and promoter engineering (Myronovskyi and Luzhetskyy 2016) have recently been applied to GC-rich actinomycetes. For instance, Gomez-Escribano *et al*. engineered *S. coelicolor* M145 strains specifically for the heterologous expression of BGCs to simplify the metabolite profiles and eliminate antimicrobial activity. This was achieved by deleting the actinorhodin, prodiginine, CPK and CDA BGCs and adding point mutations into the *rpoB* and *rpsL* genes to increase the production of secondary metabolites. The point mutations in *rpoB* and *rpsL* increased the production of **chloramphenicol** and **congocidine** by 40- and 30-fold, respectively, therefore validating the BGC-metabolite link (Gomez-Escribano and Bibb 2011). In another example, genome mining was recently used to confirm the presence of a lasso peptide BGC in the genome of a marine *Streptomyces* sp. SCSIO ZS0098 strain that was known to produce the antimicrobial type I lasso peptide **aborycin**. In this study, the utility of strain engineering was used to validate this link through the heterologous expression of the candidate aborycin BGC in *S. coelicolor* M1152 (Shao *et al*. 2019).

Genetic manipulations can also be applied in combination with genome mining to induce metabolite production. For example, genome mining of a marine *Streptomyces* strain previously known to produce anthracenes and xiamycin A also revealed an ansamycin BGC. By removing the anthracenes and xiamycin A BGCs, the mutant strain was found to produce two new napthoquinone macrolides, **olimyicn A and B** (Maansson *et al*. 2016; Sun *et al*. 2018). A conserved set of five regulatory genes previously characterised by Sidda *et al*. were used as a query to both search and identify atypical BGCs in *Streptomyces sclerotialus* NRRL ISP-5269 (Sidda *et al*. 2014; Alberti *et al*. 2019). This approach was then used to identify an atypical *scl* BGC which was transferred and heterologously expressed in *Streptomyces albus*, resulting in the production of scleric acid, a secondary metabolite with moderate activity against *Mycobacterium tuberculosis* and inhibitory activity on the cancer-associated enzyme NNMT (Alberti *et al*. 2019). An additional example of genome mining combined with heterologous expression and chemical analysis, was a study of the fungal strains *Arthrinium* sp. NF2194 and *Nectria* sp. Z14-w, which resulted in the isolation of eight new meroterpenoids, two of which exhibited immunosuppressive bioactivity (Zhang *et al*. 2018).

## CURRENT CHALLENGES

While genome mining and the application of genomic techniques have hugely benefited the genome-led secondary metabolite discovery pipeline, there are still important challenges that need to be addressed in order to maximise the potential that these approaches offer. Arguably, the bottleneck in discovery is our narrow understanding of the total biosynthetic and chemical space of microbial secondary metabolites. While prediction pipelines like SMURF (Khaldi *et al*. 2010) and antiSMASH (Medema *et al*. 2011; Blin *et al*. 2017) greatly facilitate the characterisation of BGCs, our knowledge of secondary metabolites is impeded as a result of up to 90% of BGCs being cryptic or silent (Abdelmohsen *et al*. 2015; Rutledge and Challis 2015; Baltz 2017; Machado, Tuttle and Jensen 2017). The lack of global transcriptome and translation data therefore makes it difficult to distinguish between BGCs that are transcriptionally silent and those that are actively transcribed but lack a link with their products (Jeong *et al*. 2016). A recent comparative transcriptomics study focused on four *Salinispora* strains to assess the effect of gene expression in the production of secondary metabolites. Only 13 out of the 49 BGCs were previously linked to their products, whereas the remaining were considered cryptic gene clusters. However, global transcriptome analyses at exponential and stationary phase revealed that more than half of the BGCs were in fact expressed (Amos *et al*. 2017). Further knowledge of protein translation will enable greater understanding of transcription levels and metabolite detection.

Another issue commonly encountered in secondary metabolites research is the lack of metabolite detection due to extraction constraints or the analytical technique limitations. For instance, studies have demonstrated the impact of extraction solvent on the detection of metabolites (Floros *et al*. 2016; Crüsemann *et al*. 2017). Although advances in instrument sensitivity (mass spectrometry and NMR spectroscopy) could arguably remedy problems of detection (Bouslimani *et al*. 2014), the use of limited experimental conditions can greatly impact the number and diversity of metabolites detected. These include, for example, culture conditions, media composition, growth stage and extraction solvent (Romano *et al*. 2018). If not taken into consideration, these variables could undermine the biosynthetic potential of the studied organism, complicating the linking process further.

The high rediscovery rate of molecules is another setback commonly encountered in secondary metabolite research. The efficient prioritisation of strains and extracts using combined comparative genomic and metabolomic approaches has proven to be a useful strategy to avoid this. For example, using an integrated approach of combining metabolomic and genomic techniques, Ong and co-workers identified novel metabolites with anti-quorum sensing activity from five bacterial strains isolated from subtidal marine samples (Ong *et al*. 2019). Their work is a good example of the application of molecular networking-based dereplication in the discovery of secondary metabolites. Effective dereplication greatly relies on the availability of comprehensive, curated, chemical databases and several commercially available databases to this effect are already in place, including AntiBase (Laatsch 2017), Dictionary of Natural Products (Buckingham 1994) and MarinLit (‘MarinLit’). However, data analysis using this approach is often complex and manual. The recent development of the Global Natural Products Social Molecular Networking (GNPS) platform represents a step-change that facilitates community-driven data curation, enabling open access analysis and sharing of MS/MS spectra (Wang *et al*. 2016). The expansion of such data sets will greatly facilitate our understanding of chemical space.

## FUTURE DIRECTIONS AND CONCLUDING REMARKS

Currently, researchers are biased towards the study of putative BGCs that encode variants of already known compounds or biosynthetic pathways, consequently biasing discovery towards analogues of known natural products. Efforts to overcome this include a trend towards the creation of datasets built upon increasing numbers of strains. Recently, a dataset consisting of genomic and metabolomic data for 363 bacterial strains was published and it is likely that more datasets of increasing size will become available (Navarro-Muñoz *et al*. 2018). As they do, the performance of automated linking approaches will improve.

Data sets are also likely to increase in terms of the data modalities they cover. Already, large datasets connecting bioactivity with genomics exist. For example, a recently published study linked genomic data for 224 bacterial strains (found on the leaves of *Arabidopsis* plants) to bioactivity data for the same strains (Helfrich *et al*. 2018). As high-throughput bioactivity screening becomes standard (Pye *et al*. 2017), it is likely that data sets combining metabolomics, genomics and bioactivity will become available, in turn, motivating the development of new computational techniques capable of analysing them. Crypticity of BGCs will always be a challenge in this domain. Transcriptomic analysis can indicate activity of BGCs (Amos *et al*. 2017), and the coupling of transcriptomic data with metabolomics, genomics and bioactivity is now possible. This would be particularly powerful when coupled with data generated using the OSMAC approach.

In conclusion, as data sets increase in strain coverage and modalities, increasingly advanced bioinformatics tools are required for their analysis. We believe that modern computing techniques, such as machine learning and artificial intelligence, have a key role to play in elucidating the links between genomes, transcriptomes, metabolomes and phenotypes. Computational tools to date have largely focused on modular secondary metabolites (e.g. NRPS and RIPPs), reflecting the relatively repetitive nature of their biosynthesis. The creation and continued growth of ground-truth data sets such as MiBIG (Medema *et al*. 2015) provides the necessary infrastructure for the development of tools based upon recent advances in machine learning, that are able to learn mappings between genomic information and molecular structure (as observed in mass spectrometry data). Current research is biased towards areas of the BGC space for which much is known about biosynthesis. Machine learning tools may be able to uncover patterns that help us illuminate larger, unknown areas of both the biosynthetic and chemical space.

## References

[bib1] Abdelmohsen UR, Grkovic T, Balasubramanian S et al. Elicitation of secondary metabolism in actinomycetes. Biotechnol Adv. 2015;33:798–811. 26087412 10.1016/j.biotechadv.2015.06.003

[bib2] Alberti F, Leng DJ, Wilkening I et al. Triggering the expression of a silent gene cluster from genetically intractable bacteria results in scleric acid discovery. Chem Sci. 2019;10:453–63. 30746093 10.1039/c8sc03814g PMC6335953

[bib3] Amos GCA, Awakawa T, Tuttle RN et al. Comparative transcriptomics as a guide to natural product discovery and biosynthetic gene cluster functionality. Proc Natl Acad Sci. 2017;114:E11121–30. 29229817 10.1073/pnas.1714381115 PMC5748202

[bib4] Baltz RH Gifted microbes for genome mining and natural product discovery. J Ind Microbiol Biotechnol. 2017;44:573–88. 27520548 10.1007/s10295-016-1815-x

[bib5] Bergmann S, Funk AN, Scherlach K et al. Activation of a silent fungal polyketide biosynthesis pathway through regulatory cross talk with a cryptic nonribosomal peptide synthetase gene cluster. Appl Environ Microbiol. 2010;76:8143–9. 20952652 10.1128/AEM.00683-10 PMC3008269

[bib6] Blin K, Wolf T, Chevrette MG et al. AntiSMASH 4.0 - improvements in chemistry prediction and gene cluster boundary identification. Nucleic Acids Res. 2017;45:W36–41. 28460038 10.1093/nar/gkx319 PMC5570095

[bib7] Bouslimani A, Sanchez LM, Garg N et al. Mass spectrometry of natural products: current{,} emerging and future technologies. Nat Prod Rep. 2014;31:718–29. 24801551 10.1039/c4np00044g PMC4161218

[bib8] Buckingham J Dictionary of Natural Products. Chapman and Hall/CRC, 1994.

[bib9] Chavali AK, Rhee SY Bioinformatics tools for the identification of gene clusters that biosynthesize specialized metabolites. Brief Bioinform. 2018;19:1022–34. 28398567 10.1093/bib/bbx020 PMC6171489

[bib10] Chevrette MG, Aicheler F, Kohlbacher O et al. SANDPUMA: Ensemble predictions of nonribosomal peptide chemistry reveal biosynthetic diversity across Actinobacteria. Bioinformatics. 2017;33:3202–10. 28633438 10.1093/bioinformatics/btx400 PMC5860034

[bib11] Cimermancic P, Medema MH, Claesen J et al. Insights into secondary metabolism from a global analysis of prokaryotic biosynthetic gene clusters. Cell. 2014;158:412–21. 25036635 10.1016/j.cell.2014.06.034 PMC4123684

[bib12] Coggill P, Finn RD, Bateman A Identifying protein domains with the Pfam database. Curr Protocols Bioinform. 2008, DOI:10.1002/0471250953.bi0205s23. 18819075

[bib13] Crüsemann M, O'Neill EC, Larson CB et al. Prioritizing natural product diversity in a collection of 146 bacterial strains based on growth and extraction protocols. J Nat Prod. 2017;80:588–97. 28335604 10.1021/acs.jnatprod.6b00722 PMC5367486

[bib14] Doroghazi JR, Albright JC, Goering AW et al. Aroadmap for natural product discovery based on large-scale genomics and metabolomics. Nat Chem Biol. 2014;10:963–8. 25262415 10.1038/nchembio.1659 PMC4201863

[bib15] Duncan KR, Crüsemann M, Lechner A et al. Molecular networking and pattern-based genome mining improves discovery of biosynthetic gene clusters and their products from salinispora species. Chem Biol. 2015;22:460–71. 25865308 10.1016/j.chembiol.2015.03.010 PMC4409930

[bib16] Floros DJ, Jensen PR, Dorrestein PC et al. A metabolomics guided exploration of marine natural product chemical space. Metabolomics. 2016;12:135. 10.1007/s11306-016-1087-5 PMC5556696 28819353

[bib17] Goering AW, McClure RA, Doroghazi JR et al. Metabologenomics: Correlation of microbial gene clusters with metabolites drives discovery of a nonribosomal peptide with an unusual amino acid monomer. ACS Cent Sci. 2016;2:299–108. 10.1021/acscentsci.5b00331 PMC4827660 27163034

[bib18] Gomez-Escribano JP, Bibb MJ Engineering Streptomyces coelicolor for heterologous expression of secondary metabolite gene clusters. Microb Biotechnol. 2011;4: DOI:10.1111/j.1751-7915.2010.00219.x. PMC3818861 21342466

[bib19] Gubbens J, Zhu H, Girard G et al. Natural product proteomining, a quantitative proteomics platform, allows rapid discovery of biosynthetic gene clusters for different classes of natural products. Chem Biol. 2014;21:707–18. 24816229 10.1016/j.chembiol.2014.03.011

[bib20] Gust B, Challis GL, Fowler K et al. PCR-targeted Streptomyces gene replacement identifies a protein domain needed for biosynthesis of the sesquiterpene soil odor geosmin. Proc Natl Acad Sci. 2003;100:1541–6. 12563033 10.1073/pnas.0337542100 PMC149868

[bib21] Helfrich EJN, Vogel CM, Ueoka R et al. Bipartite interactions, antibiotic production and biosynthetic potential of the Arabidopsis leaf microbiome. Nat Microbiol. 2018;3:909–19. 30038309 10.1038/s41564-018-0200-0 PMC7115891

[bib22] Huo L, Hug JJ, Fu C et al. Heterologous expression of bacterial natural product biosynthetic pathways. Nat Prod Rep. 2019, DOI:10.1039/c8np00091c. 30620035

[bib23] Jeong Y, Kim JN, Kim MW et al. The dynamic transcriptional and translational landscape of the model antibiotic producer Streptomyces coelicolor A3(2). Nat Commun. 2016;7:11605. 27251447 10.1038/ncomms11605 PMC4895711

[bib24] Johnston CW, Skinnider MA, Wyatt MA et al. An automated Genomes-to-Natural Products platform (GNP) for the discovery of modular natural products. Nat Commun. 2015;6, DOI:10.1038/ncomms9421. PMC4598715 26412281

[bib25] Kaweewan I, Komaki H, Hemmi H et al. Isolation and structure determination of new antibacterial peptide curacomycin based on genome mining. Asian J Org Chem. 2017;6:1838-44, DOI:10.1002/ajoc.201700433.

[bib26] Kaweewan I, Komaki H, Hemmi H et al. Isolation and structure determination of a new cytotoxic peptide, curacozole, from Streptomyces curacoi based on genome mining. J Antibiot (Tokyo). 2019;72:1–7. 30310179 10.1038/s41429-018-0105-4

[bib27] Kersten RD, Yang YL, Xu Y et al. A mass spectrometry-guided genome mining approach for natural product peptidogenomics. Nat Chem Biol. 2011;7:794–802. 21983601 10.1038/nchembio.684 PMC3258187

[bib28] Khaldi N, Seifuddin FT, Turner G et al. SMURF: Genomic mapping of fungal secondary metabolite clusters. Fungal Genet Biol. 2010;47:736–41. 20554054 10.1016/j.fgb.2010.06.003 PMC2916752

[bib29] Laatsch H AntiBase: The Natural Compound Identifier. Wiley-Vch. 2017. ISBN:978-3-527-33841-2

[bib30] Lin K, Zhu L, Zhang DY An initial strategy for comparing proteins at the domain architecture level. Bioinformatics. 2006;22:2081–6. 16837531 10.1093/bioinformatics/btl366

[bib31] Maansson M, Vynne NG, Klitgaard A et al. An integrated metabolomic and genomic mining workflow to uncover the biosynthetic potential of bacteria. mSystems. 2016;1:e00028–15. 10.1128/mSystems.00028-15 PMC5069768 27822535

[bib32] Machado H, Tuttle RN, Jensen PR Omics-based natural product discovery and the lexicon of genome mining. Curr Opin Microbiol. 2017;39:136–42. 29175703 10.1016/j.mib.2017.10.025 PMC5732065

[bib34] McClure RA, Goering AW, Ju K-S et al. Elucidating the rimosamide-detoxin natural product families and their biosynthesis using metabolite/gene cluster correlations. ACS Chem Biol. 2016;11:3452–60. 27809474 10.1021/acschembio.6b00779 PMC5295535

[bib35] Medema MH, Blin K, Cimermancic P et al. AntiSMASH: Rapid identification, annotation and analysis of secondary metabolite biosynthesis gene clusters in bacterial and fungal genome sequences. Nucl Acids Res. 2011;39:W339–46. 21672958 10.1093/nar/gkr466 PMC3125804

[bib36] Medema MH, Kottmann R, Yilmaz P et al. Minimum information about a biosynthetic gene cluster. Nat Chem Biol. 2015;11:625. 26284661 10.1038/nchembio.1890 PMC5714517

[bib37] Medema MH, Paalvast Y, Nguyen DD et al. Pep2Path: automated mass spectrometry-guided genome mining of peptidic natural products. PLOS Comput Biol. 2014;10:e1003822. 25188327 10.1371/journal.pcbi.1003822 PMC4154637

[bib38] Médigue C, Krin E, Pascal G et al. Coping with cold: The genome of the versatile marine Antarctica bacterium Pseudoalteromonas haloplanktis TAC125. Genome Res. 2005;15:1325–35. 16169927 10.1101/gr.4126905 PMC1240074

[bib39] Mohimani H, Gurevich A, Mikheenko A et al. Dereplication of peptidic natural products through database search of mass spectra. Nat Chem Biol. 2017;13:30–37. 27820803 10.1038/nchembio.2219 PMC5409158

[bib40] Mohimani H, Gurevich A, Shlemov A et al. Dereplication of microbial metabolites through database search of mass spectra. Nat Commun. 2018;9:4035. 30279420 10.1038/s41467-018-06082-8 PMC6168521

[bib41] Mohimani H, Kersten RD, Liu WT et al. Automated genome mining of ribosomal peptide natural products. ACS Chem Biol. 2014a;9:1545–51. 24802639 10.1021/cb500199h PMC4215869

[bib42] Mohimani H, Liu WT, Kersten RD et al. NRPquest: Coupling mass spectrometry and genome mining for nonribosomal peptide discovery. J Nat Prod. 2014b;77:1902–9. 25116163 10.1021/np500370c PMC4143176

[bib43] Myronovskyi M, Luzhetskyy A Native and engineered promoters in natural product discovery. Nat Prod Rep. 2016;33:1006–19. 27438486 10.1039/c6np00002a

[bib44] Navarro-Muñoz J, Selem-Mojica N, Mullowney M et al. A computational framework for systematic exploration of biosynthetic diversity from large-scale genomic data. bioRxiv. 2018, DOI:10.1101/445270.

[bib45] Nguyen DD, Wu C-H, Moree WJ et al. MS/MS networking guided analysis of molecule and gene cluster families. Proc Natl Acad Sci. 2013;110:E2611–20. 23798442 10.1073/pnas.1303471110 PMC3710860

[bib46] Ong JFM, Goh HC, Lim SC et al. Integrated genomic and metabolomic approach to the discovery of potential anti-quorum sensing natural products from microbes associated with marine samples from Singapore. Mar Drugs. 2019;17:E72. 30669697 10.3390/md17010072 PMC6356914

[bib47] Panter F, Krug D, Müller R Novel methoxymethacrylate natural products uncovered by statistics-based mining of the myxococcus fulvus secondary metabolome. ACS Chem Biol. 2019;14:88–98. 30543288 10.1021/acschembio.8b00948

[bib48] Parkinson EI, Tryon JH, Goering AW et al. Discovery of the tyrobetaine natural products and their biosynthetic gene cluster via metabologenomics. ACS Chem Biol. 2018;13:1029–37. 29510029 10.1021/acschembio.7b01089 PMC5944846

[bib49] Pye CR, Bertin MJ, Lokey RS et al. Retrospective analysis of natural products provides insights for future discovery trends. Proc Natl Acad Sci. 2017;114:5601–6. 28461474 10.1073/pnas.1614680114 PMC5465889

[bib50] Romano S, Jackson SA, Patry S et al. Extending the “one strain many compounds” (OSMAC) principle to marine microorganisms. Mar Drugs. 2018;16:E244. 30041461 10.3390/md16070244 PMC6070831

[bib51] Rutledge PJ, Challis GL Discovery of microbial natural products by activation of silent biosynthetic gene clusters. Nat Rev Microbiol. 2015;13:509–23. 26119570 10.1038/nrmicro3496

[bib52] Schneider O, Simic N, Aachmann FL et al. Genome Mining of Streptomyces sp. YIM 130001 Isolated From Lichen Affords New Thiopeptide Antibiotic. Front Microbiol. 2018;9:3139. 30619207 10.3389/fmicb.2018.03139 PMC6306032

[bib53] Shao M, Ma J, Li Q et al. Identification of the anti-infective aborycin biosynthetic gene cluster from deep-sea-derived streptomyces sp. SCSIO ZS0098 enables production in a heterologous host. Mar Drugs. 2019;17:E127. 30795576 10.3390/md17020127 PMC6409603

[bib54] Sidda JD, Song L, Poon V et al. Discovery of a family of γ-aminobutyrate ureas via rational derepression of a silent bacterial gene cluster. Chem Sci. 2014;5:86–9., DOI:10.1039/c3sc52536h.

[bib55] Skinnider MA, Dejong CA, Rees PN et al. Genomes to natural products PRediction Informatics for Secondary Metabolomes (PRISM). Nucl Acids Res. 2015;43:9645–62. 26442528 10.1093/nar/gkv1012 PMC4787774

[bib56] Skinnider MA, Merwin NJ, Johnston CW et al. PRISM 3: Expanded prediction of natural product chemical structures from microbial genomes. Nucl Acids Res. 2017;45:W49–54. 28460067 10.1093/nar/gkx320 PMC5570231

[bib57] Son S, Jang M, Lee B et al. Ulleungdin, a Lasso Peptide with Cancer Cell Migration Inhibitory Activity Discovered by the Genome Mining Approach. J Nat Prod. 2018;81:2205–11., DOI:10.1021/acs.jnatprod.8b00449. 30251851

[bib58] Sun C, Zhang C, Qin X et al. Genome mining of Streptomyces olivaceus SCSIO T05: Discovery of olimycins A and B and assignment of absolute configurations. Tetrahedron. 2018;74:199–203.

[bib59] Takasaka N, Kaweewan I, Ohnishi-Kameyama M et al. Isolation of a new antibacterial peptide actinokineosin from Actinokineospora spheciospongiae based on genome mining. Lett Appl Microbiol. 2017;64:150–7. 27813109 10.1111/lam.12693

[bib60] Tao W, Yang A, Deng Z et al. CRISPR/Cas9-based editing of streptomyces for discovery, characterization, and production of natural products. Front Microbiol. 2018;9:1660. 30087666 10.3389/fmicb.2018.01660 PMC6066502

[bib61] Thomas T, Evans FF, Schleheck D et al. Analysis of the Pseudoalteromonas tunicata Genome Reveals Properties of a Surface-Associated Life Style in the Marine Environment. PLoS One. 2008;3:1–11. 10.1371/journal.pone.0003252 PMC2536512 18813346

[bib62] Tietz JI, Schwalen CJ, Patel PS et al. A new genome-mining tool redefines the lasso peptide biosynthetic landscape. Nat Chem Biol. 2017;13:470–8. 28244986 10.1038/nchembio.2319 PMC5391289

[bib63] Ueoka R, Bhushan A, Probst SI et al. Genome-based identification of a plant-associated marine bacterium as a rich natural product source. Angew Chemie - Int Ed. 2018;57:14519–23. 10.1002/anie.201805673 30025185

[bib64] Umemura M, Koike H, Machida M Motif-independent de novo detection of secondary metabolite gene clusters-toward identification from filamentous fungi. Front Microbiol. 2015;6:371, DOI:10.3389/fmicb.2015.00371. 25999925 PMC4419862

[bib65] Umemura M, Koike H, Nagano N et al. MIDDAS-M: Motif-independent de novo detection of secondary metabolite gene clusters through the integration of genome sequencing and transcriptome data. PLoS One. 2013;8:e84028. 24391870 10.1371/journal.pone.0084028 PMC3877130

[bib66] Wang M, Carver JJ, Phelan V V et al. Sharing and community curation of mass spectrometry data with Global Natural Products Social Molecular Networking. Nat Biotechnol. 2016;34:828–37. 27504778 10.1038/nbt.3597 PMC5321674

[bib67] Xu B, Aitken EJ, Baker BP et al. Genome mining, isolation, chemical synthesis and biological evaluation of a novel lanthipeptide, tikitericin, from the extremophilic microorganism: Thermogemmatispora strain T81. Chem Sci. 2018;9:7311–7. 30294420 10.1039/c8sc02170h PMC6167946

[bib68] Zhang X, Wang TT, Xu QL et al. Genome Mining and Comparative Biosynthesis of Meroterpenoids from Two Phylogenetically Distinct Fungi. Angew Chemie - Int Ed. 2018;57:8184–8. 10.1002/anie.201804317 29797385

